# The relationship between serum astroglial and neuronal markers and AQP4 and MOG autoantibodies

**DOI:** 10.1186/s12014-024-09466-9

**Published:** 2024-04-05

**Authors:** Miyo K. Chatanaka, Lisa M. Avery, Maria D. Pasic, Shanthan Sithravadivel, Dalia Rotstein, Catherine Demos, Rachel Cohen, Taron Gorham, Mingyue Wang, Martin Stengelin, Anu Mathew, George Sigal, Jacob Wohlstadter, Ioannis Prassas, Eleftherios P. Diamandis

**Affiliations:** 1 Department of Laboratory and Medicine Pathobiology, University of Toronto, 60 Murray St. Box 32, Floor 6, Rm L6-201, Toronto, ON M5T 3L9 Canada; 2 Laboratory Medicine Program, University Health Network, Toronto, ON Canada; 3 Biostatistics Division, Dalla Lana School of Public Health, University of Toronto, Toronto, Canada; 4 Department of Biostatistics, The Princess Margaret Cancer Centre, University of Toronto, Toronto, Canada; 5 Department of Laboratory Medicine, St. Joseph’s Health Centre, Unity Health Toronto, Toronto, Canada; 6 St. Michael’s Hospital, Toronto, ON M5B 1W8 Canada; 7 Meso Scale Diagnostics, LLC, Rockville, MD USA; 8 Lunenfeld- Tanenbaum Research Institute, Mount Sinai Hospital, Toronto, ON Canada

**Keywords:** Aquaporin 4, Myelin oligodendrocyte glycoprotein, Autoantibody, Autoimmunity, Glial fibrillary acidic protein, Tau, Neurofilament-light

## Abstract

**Background:**

Certain demyelinating disorders, such as neuromyelitis optica spectrum disorder (NMOSD) and myelin oligodendrocyte glycoprotein antibody-associated disease (MOGAD) exhibit serum autoantibodies against aquaporin-4 (αAQP4) and myelin oligodendrocyte glycoprotein (αMOG). The variability of the autoantibody presentation warrants further research into subtyping each case.

**Methods:**

To elucidate the relationship between astroglial and neuronal protein concentrations in the peripheral circulation with occurrence of these autoantibodies, 86 serum samples were analyzed using immunoassays. The protein concentration of glial fibrillary acidic protein (GFAP), neurofilament light chain (NFL) and tau protein was measured in 3 groups of subcategories of suspected NMOSD: αAQP4 positive (*n* = 20), αMOG positive (*n* = 32) and αMOG/αAQP4 seronegative (*n* = 34). Kruskal-Wallis analysis, univariate predictor analysis, and multivariate logistic regression with ROC curves were performed.

**Results:**

GFAP and NFL concentrations were significantly elevated in the αAQP4 positive group (*p* = 0.003; *p* = 0.042, respectively), and tau was elevated in the αMOG/αAQP4 seronegative group (*p* < 0.001). A logistic regression model to classify serostatus was able to separate αAQP4 seropositivity using GFAP + tau, and αMOG seropositivity using tau. The areas under the ROC curves (AUCs) were 0.77 and 0.72, respectively. Finally, a combined seropositivity versus negative status logistic regression model was generated, with AUC = 0.80.

**Conclusion:**

The 3 markers can univariately and multivariately classify with moderate accuracy the samples with seropositivity and seronegativity for αAQP4 and αMOG.

**Supplementary Information:**

The online version contains supplementary material available at 10.1186/s12014-024-09466-9.

## Introduction

The existence of autoantibodies against glial cell proteins, namely aquaporin-4 (αAQP4) and myelin oligodendrocyte glycoprotein (αMOG) is an important pathobiological feature of certain central nervous system (CNS) autoimmune demyelinating diseases, such as neuromyelitis optica spectrum disorder (NMOSD) and myelin oligodendrocyte glycoprotein antibody-associated disease (MOGAD) [1–3]. According to the 2015 diagnostic criteria [4], these diseases are: (i) characterized by longitudinally extensive transverse myelitis (TM), optic neuritis (ON), and brainstem dysfunction, among other symptoms; (ii) they are positive for αAQP4 and/or αMOG; and (iii) are a distinct entity from multiple sclerosis (MS) [5–7], thus requiring its exclusion. The symptoms can occur simultaneously, or can present in a limited form (e.g., isolated ON) [8], and for MOGAD, the presence of αMOG is a requirement for diagnosis [9].

Intriguingly, however, not all patients with NMOSD are positive for the above specified autoantibodies (these are known as seronegative patients), thus posing challenges in the diagnosis, prognosis and treatment of the disorder [8]. In addition, markers to predict a monophasic or relapsing disease course are lacking, as well as predictors of treatment response [8]. In certain circumstances, testing for autoantibodies in the cerebrospinal fluid (CSF), in addition to serum, is necessary since some studies have reported autoantibody positivity only in the CSF of patients with a MOGAD phenotype [10].

Biomarkers are of paramount importance in efforts to: (i) elucidate differentiation markers between NMOSD and MOGAD (and MS), (ii) assist in disease prognosis and treatment response, (iii) further understand the intra-NMOSD patient variability, (iv) determine the relapse risk, and (v) establish methods to evaluate disease severity [11]. In particular, markers of neuronal and astroglial damage, including glial fibrillary acidic protein (GFAP) [12–15], neurofilament light chain (NFL, also known as NEFL, NF-L, NfL) [16, 17] and tau [18] can shed light into relapse risk versus a monophasic course, predict treatment response and disease severity [8].

In this paper, we focus on elucidating differentiation markers between αAQP4 and αMOG positive and negative samples (αAQP4 positive = αAQP4+; αMOG positive = αMOG+; double seronegative = αMOG-/αAQP4-), and whether these markers can predict autoantibody serostatus. We did not encounter any double seropositive patients in our cohort. We tested serum samples from patients with suspected NMOSD that had recently been tested for αAQP4 and αMOG presence. We also quantified the protein concentrations (not the autoantibodies) of GFAP, NFL and tau in serum. We then examined the possible relationship between αAQP4 and αMOG with the serum markers of neuronal and astrocytic injury, namely the proteins GFAP, NFL and tau.

## Materials and methods

### Sample collection and analysis

Serum samples from suspected NMOSD patients were provided by the Unity Health Toronto Immunology Laboratory, Toronto, Canada, under institutional Review Board approval (ethical approval number: #19-0321-E). Patients provided a written informed consent for this study. The samples were sent to Unity Health Toronto by province of Ontario-wide third-party laboratories, for the purpose of testing them for αAQP4 and αMOG, as one of the diagnostic requirements for suspected NMOSD. The suspected NMOSD serum samples were divided into three categories: αAQP4+ (*n* = 20), αMOG+ (*n* = 32) and αMOG-/αAQP4- (*n* = 34). There were no samples positive for both αAQP4 and αMOG.

All blood samples were collected under standard laboratory procedures, centrifuged at 3,000 x g after a 30 min coagulation at room temperature, and serum was stored at -80 °C in polypropylene tubes. Sera were then aliquoted, coded and stored at Mount Sinai Hospital, Toronto, Canada at -80 °C until processing.

An aliquot of each sample (*n* = 86) was coded with a unique identification number and transferred on dry ice to Meso Scale Discovery (MSD, a division of Meso Scale Diagnostics, LLC.; Rockville, MD, USA) for testing, using a 3-marker ultrasensitive electrochemiluminescence sandwich immunoassay (GFAP, NFL and tau). MSD was blinded regarding the identity of the samples and the code was broken after analysis was completed. The code connecting patient and sample identity was known only to the principal investigator (EP Diamandis).

### MSD® assays

A new custom multiplex ultrasensitive immunoassay based on electrochemiluminescence detection was used to measure GFAP, tau and NFL in a 96-well plate format. This panel is now commercially available: S-PLEX® Neurology Panel 1 (Meso Scale Discovery, Rockville, MD; catalog # K15640S). The analytical sensitivities of the 3 protein assays (GFAP, NFL, tau) were 0.19pg/mL, 1.28pg/mL and 0.04pg/mL respectively, and their precision was < 15%. The assay requires 25 µL of two-fold diluted serum or plasma. The MSD website (www.mesoscale.com) and our previous publications [19, 20] provide additional information about this assay technology.

### Assays at unity health Toronto

Serum samples were analyzed for immunoglobulin class IgG against AQP4 and/or MOG using a semiquantitative in vitro commercial kit (EUROIMMUN Indirect Immunofluoresence Test- IIFT; FA 1128-1005-1, FA 1128-1010-1), according to manufacturer’s recommendations. The samples were diluted ten-fold in a provided buffer and the positivity was measured through fluorescence pattern intensity (graded as 0–5). There is no upper limit to the measurement range of this kit. The manufacturer-specified analytical sensitivity and specificity for the αAQP4 IIFT were 75% and 99.9%, respectively. Similarly, for the αMOG IIFT, the analytical sensitivity and specificity were 95% and 84.9%, respectively.

### Statistical analysis

**Fig. 1:**
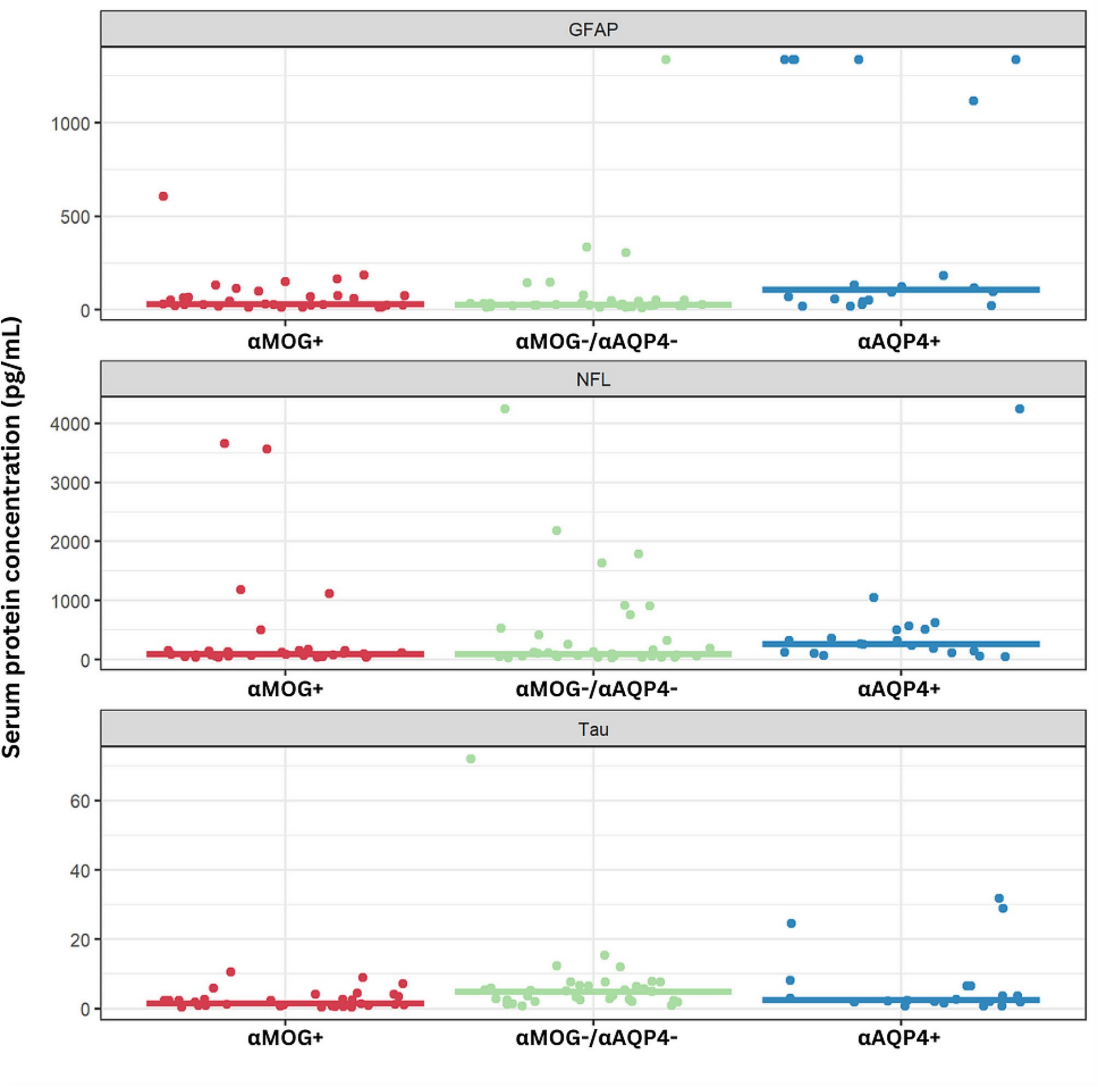
Scatterplots of protein concentrations of the 3 biomarkers analyzed in the αAQP4+, αMOG+ and αMOG-/αAQP4-groups. The horizontal lines represent the median of each group, and each dot represents the value of the marker in an individual sample. For numerical values and *P*-values, see Table 1

Statistical analyses were performed using R (version 4.2.3 [21]). The concentration values of GFAP, NFL and tau were *natural log*-transformed after histograms of the 3 markers revealed skewed distributions (see Supplementary Information section, Fig. 1), and the descriptive statistics were reported. Non-parametric Kruskal-Wallis tests were applied to each biomarker, to determine if the median varied across diagnostic groups, and the *P*-values were adjusted for false discovery rate (FDR) (Table 1).

**Table 1:** Descriptive statistics for the 3 markers in the αMOG+, αMOG-/αAQP4- and αAQP4 + groups

Covariate (pg/mL)	αMOG+ (*n* = 32)^2^	αMOG-/αAQP4- (*n* = 34)	αAQP4+ (*n* = 20)	***P***-value^3^	αAQP4+ vs. αMOG-/αAQP4- (***P***-value)^4^	αAQP4+ vs. αMOG+ (***P***-value)^4^	αMOG+ vs. αMOG-/αAQP4- (***P***-value)^4^
**GFAP**				**0.003**			
**Dunn Z test**					**3.4 (< 0.001)**	**2.6** **(0.005)**	**-0.9** **(0.17)**
Mean (sd)^1^	73.5 (108)	92.1 (232)	443 (579)				
Median (Min, Max)	30.8 (12.5, 607)	28.1 (9.8, 1338)	107.7 (19.4, 1338)				
**NFL**				**0.042**			
**Dunn Z test**					**2.2** **(0.015)**	**2.4 (0.009)**	**0.3** **(0.4)**
Mean (sd)	390 (886)	462 (859)	506 (912)				
Median (Min, Max)	93.6 (36.5, 3658)	99.6 (32.5, 4240)	265 (49.5, 4240)				
**tau**				**< 0.001**			
**Dunn Z test**					**-1.3** **(0.1)**	**1.9 (0.026)**	**3.7** **(< 0.001)**
Mean (sd)	2.5 (2.5)	7.0 (12.0)	6.8 (9.6)				
Median (Min, Max)	1.6 (0.4, 10.5)	5.0 (0.7, 72.0)	2.5 (0.7, 31.8)				

^1^sd = standard deviation

^2^n = number of samples

^3^*P*-value was calculated by the Kruskal-Wallis test

^4^*P*-value was calculated by the Dunn’s Post-Hoc test

In parallel, univariate logistic regression models were used on the transformed values to determine if the 3 serum markers were associated with αMOG or αAQP4 status (Table 2). Exploratory multiple logistic regression was used to predict cases that were either αMOG+ or αAQP4+ from combinations of GFAP, tau and NFL. Performance metrics and a receiver operating characteristic (ROC) curve with the area under the curve (AUC) were calculated. To provide an estimate of the AUC that may be expected on an uncharacterized sample, a robust internal validation process was used to calculate the optimism-adjusted AUC [22, 23].

**Table 2:** GFAP, NFL and tau as univariate predictors of autoantibody positivity

Autoantibody status			
*αAQP4 Status*			
Marker	OR (95% CI)^1^	***P***-value^2^	Number of samples
GFAP	2.18 (1.45, 3.51)	< 0.001	20
NFL	1.43 (0.97, 2.11)	0.067	
tau	1.23 (0.76, 2.00)	0.4	
*αMOG Status*			
GFAP	0.78 (0.52, 1.13)	0.21	32
NFL	0.78 (0.52, 1.12)	0.19	
tau	0.37 (0.20, 0.63)	< 0.001	

^1^OR: Odds ratio; CI: confidence interval

^2^*P*-value was calculated by logistic regression

## Results

Median protein concentrations varied significantly across αMOG/αAQP4 samples (Table 1). αAQP4+ sera had the highest mean and median values for GFAP and NFL, whereas αMOG-/αAQP4- had the highest mean and median values for tau (Table 1; Fig. 1). αMOG+ had the lowest tau values (Table 1; Fig. 1). Figure 1 represents scatterplots of the protein concentrations in pg/mL in the 3 groups. The αAQP4+ group had higher median NFL values, but the αMOG-/αAQP4- group had some samples with moderately high NFL concentrations.

**Fig. 2:**
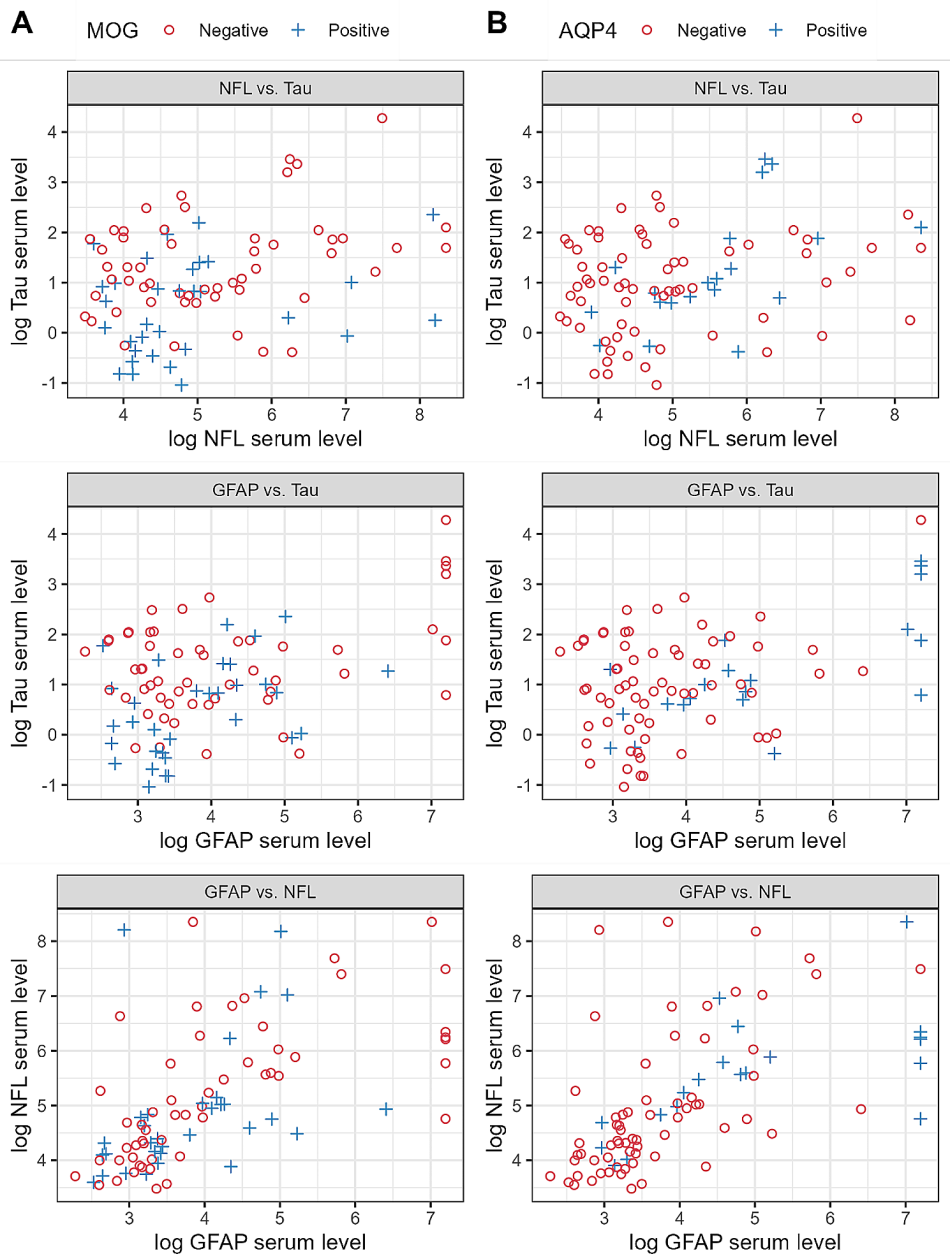
Pairwise plots of the 3 markers: GFAP, NFL and tau. The x and y axes represent the logarithmic protein serum values. **(A)** αMOG status, **(B)** αAQP4 status

**Fig. 3:**
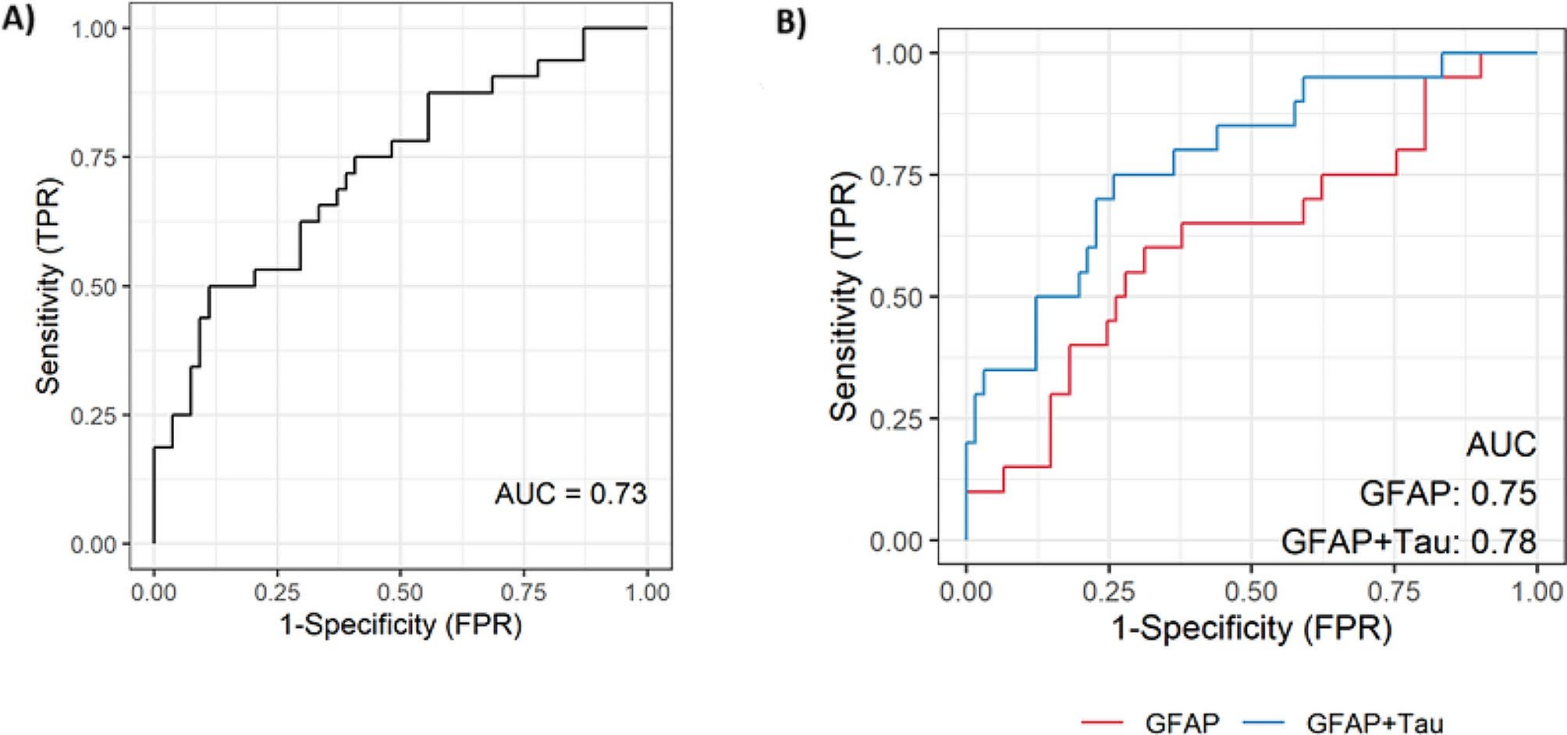
**(A)** ROC curve for predicting αMOG+ from tau values. The bootstrapped optimism-adjusted AUC is 0.72 (unadjusted value = 0.73). The AUC values from combining NFL + tau or GFAP + tau were lower than for tau alone (data not shown). **(B)** ROC curves for predicting αAQP4+ status using GFAP (red) and GFAP + tau (blue). The non-adjusted and bootstrapped AUC values of GFAP alone are 0.75 (adjusted) and 0.75 (non-adjusted) and for GFAP + tau are 0.78 and 0.77, respectively

Figure 2 depicts pairwise plots for each pair of proteins for the two autoantibody statuses, to determine if samples from the diagnostic groups could be differentiated based on any two proteins. Due to the relatively small number of samples in each category, and the significant overlap, the presented data allow only qualitative observations based on the graphs of Fig. 2. Figure 2 (A) shows that the combination of NFL and tau (Fig. 2 (A), top left panel) seems to aggregate most of the αMOG+ to the bottom left (low concentrations for both proteins). Interestingly, however, the optimism-adjusted AUC of the tau model was 0.72 (Fig. 3 (A)) and for the NFL + tau was 0.71 (Table 3), showing that tau alone is a slightly better classifier than the NFL + tau combination. Figure 2 (B), lower panel shows that there is a subset of αAQP4+ samples that can be distinguished from the rest of the samples, with high GFAP (higher than 7 in log serum value). In addition, from an exploratory multivariable logistic regression analysis, GFAP seems to be the strongest predictor of αAQP4+ for both GFAP + NFL (Fig. 2 (B), panel bottom left) and GFAP + tau models (Fig. 2 (B), top right panel), both resulting in AUCs of 0.77, despite NFL and tau not being significant by themselves (Table 3).

**Table 3:** GFAP, NFL and tau as exploratory multivariable predictors of αAQP4 and αMOG positivity

Autoantibodies					
*αAQP4*					
Markers		OR (95% CI)^1^	***P***-value^2^	Number of samples	Adjusted^3^ AUC
GFAP + NFL	GFAP	2.37 (1.37, 4.11)	0.002	20	0.77
	NFL	0.87 (0.50, 1.51)	0.62		
GFAP + tau	GFAP	2.56 (1.51, 4.31)	< 0.001	20	0.77
	tau	0.69 (0.38, 1.27)	0.24		
*αMOG*					
tau + NFL	tau	0.38 (0.21, 0.67)	< 0.001	32	0.71
	NFL	0.94 (0.62, 1.43)	0.78		
tau + GFAP	tau	0.37 (0.21, 0.66)	< 0.001	32	0.71
	GFAP	1.00 (0.63, 1.57)	0.99		

^1^OR: Odds ratio; CI: confidence interval; AUC: area under the ROC curve

^2^*P*-value was calculated by multiple logistic regression

^3^Optimism-adjusted

Figure 3 depicts the ROC curves for (A) the αMOG status (positive or negative) based on tau as a discriminator, and (B) the αAQP4 status based on GFAP alone and GFAP + tau combination. The calculated AUCs in each subfigure represent the non-adjusted values, while bootstrap-adjusted values were 0.01 units smaller. In Fig. 3 (A), although not corrected for over-fitting, the sensitivity of the model at optimal tau cutoff was 0.5 (0.32, 0.68 CI), the specificity 0.87 (0.75, 0.95 CI), the positive predictive value 0.70 (0.47, 0.87 CI), the negative predictive value 0.75 (0.62, 0.85 CI) and the accuracy 0.73 (0.63, 0.82 CI). Similarly, in Fig. 3 (B), the combined model (GFAP + tau) exhibited a better sensitivity of 0.35 (0.15, 0.59 CI) versus 0.30 (0.12, 0.54 CI) for GFAP alone. For the combined GFAP + tau model, at optimal cutoffs, the specificity was 0.94 (0.85, 0.98 CI), the positive predictive value was 0.64 (0.31, 0.89 CI), the negative predictive value was 0.83 (0.72, 0.90 CI) and the accuracy was 0.80 (0.70, 0.88 CI).

**Fig. 4:**
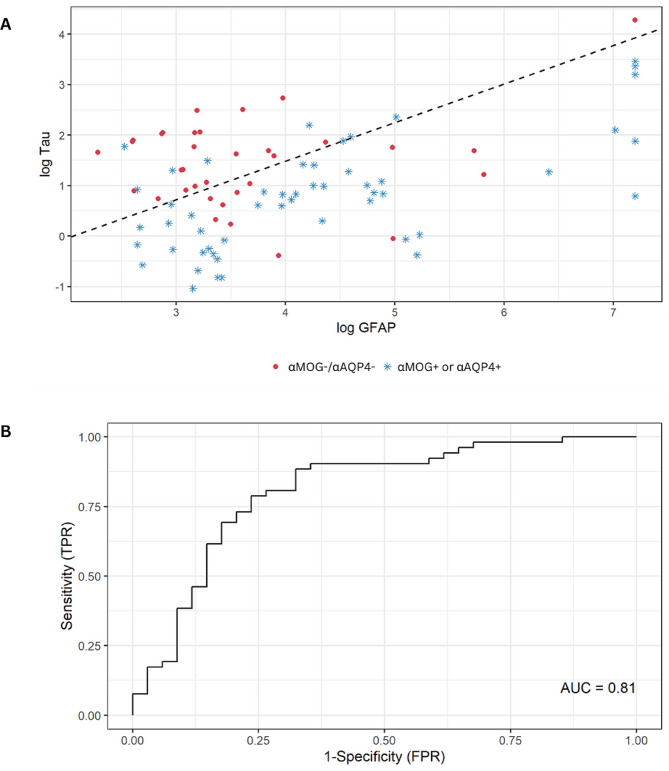
Multivariable logistic regression was used to separate the cases with αAQP4+ or αMOG+ from the cases with a double negative status. **(A)** The scatterplot of the log-transformed GFAP vs. tau. The dashed line indicates the prediction of αAQP4+ or αMOG+ versus αMOG-/αAQP4-. **(B)** The ROC curve has an unadjusted AUC of 0.81 (0.80 after optimism-adjustment)

A multivariable logistic regression model was also developed to separate the samples with positive autoantibody status from the double-negative status based on GFAP and tau, after the Kruskal-Wallis test showed significantly different medians for both markers (*p* < 0.001). Figure 4 depicts a binary classification (either autoantibody positive or negative), with (A) showing a scatterplot of the log transformed GFAP versus tau values, and (B) showing the ROC curve for this binary classification (using GFAP and tau), with an AUC = 0.81 (adjusted 0.80). Particularly for (A), at optimal cutoffs, the sensitivity of the model is 0.87 (0.74, 0.94 CI), the specificity 0.68 (0.49, 0.83 CI), the positive predictive value 0.80 (0.68, 0.90 CI), the negative predictive value 0.77 (0.58, 0.90 CI) and the accuracy 0.79 (0.69, 0.87 CI).

## Discussion

Markers of neuronal and astroglial damage are indicative of CNS injury, and they are released into the CSF with subsequent leakage in the periphery, where they can be quantified [8, 24, 25]. In NMOSD, this is hypothesized to occur with the timing of sampling, with relapsing episodes showing high concentration of these markers, while during remission there are lower levels [8].

In this study, we aimed to examine the possible relationship between αAQP4 and αMOG with GFAP, NFL and tau, to find complementary biomarkers of differentiation in the αAQP4+, αMOG+ and αMOG-/αAQP4- groups in samples from patients with suspected NMOSD. The clear separation of the patient groups is crucial, since inappropriate treatments following a false diagnosis may exacerbate symptoms [11, 26–28]. With current assays showing low sensitivity for αAQP4 [29] and being dependent on treatment and clinical status [30, 31], and better, newer assays not being used in the clinic as of yet [32], strong interrelated differentiators should be useful.

Several studies in αAQP4+, αMOG+ and αMOG-/αAQP4- NMOSD patients have previously analyzed GFAP, NFL and tau in serum [33–36]. GFAP is the main cytoskeletal filamentous protein of mature astrocytes, involved in supporting the glial cell structure and strength, as well as supporting neurons and the Blood-Brain Barrier (BBB) [13, 37]. During astrogliosis or brain-related injury, GFAP is excreted into the circulation [12, 24], thus being a good biomarker of traumatic brain injury (TBI) [38], MS [39, 40], dementia [41, 42], brain tumors [43–45], and other neurological diseases [46]. NFL, along with other neurofilament proteins, is an intracellular protein in neurons that partakes in axonal stability and radial growth, and it is released after neuroaxonal damage [17]. Just like GFAP, NFL is a promising biomarker of neurodegeneration in MS [17, 47], dementia [48], TBI [38, 49], amyotrophic lateral sclerosis (ALS) and other neurological diseases [48, 50]. NFL is especially important in its ability to reflect ongoing axonal degeneration, thus shedding light on the pathophysiology of NMOSD and its subcategorizations [38]. Lastly, tau is a microtubule-associated protein that is important in neuronal health and function, with multiple alterations being seen in disease [51, 52]. Aberrant tau forms deposited in the blood (and CSF) are a biomarker of tauopathies, Alzheimer’s disease (AD) and other dementias [51, 53, 54], Creutzfeldt-Jakob disease [55] and other brain related neuropathies [56, 57].

In our study, we found that GFAP is significantly higher in αAQP4+ samples compared to αMOG+ and αMOG-/αAQP4- (Fig. 1; Table 1). The one sample in the αMOG-/αAQP4- group that exhibits high GFAP concentration (1,338 pg/mL) could be attributed to: (i) the patient having recently experienced a neurological attack, (ii) the elevated GFAP being a marker of a future episode, or (iii) they had recently experienced an independent TBI event. Importantly, GFAP concentration was a significant univariate predictor of αAQP4 status (Table 2), and GFAP concentration was significant in the multivariable logistic regression for αAQP4 status (Table 3). It was also used as part of the model to classify samples based on their autoantibody seropositivity, with an AUC of 0.80 after optimism-adjustment (Fig. 4). The detection of high GFAP in αAQP4 + samples provides insights into the molecular pathomechanism of NMOSD, and it hints at αAQP4 causing extensive astroglial damage and/or astrogliosis, which later drains into the circulation through arachnoid villi, the glymphatic system or the BBB and blood-CSF barriers [12].

There are a few clinical studies that have analyzed GFAP in serum of patients with NMOSD, specifically with αAQP4 status, reporting conflicting results. In the first study, GFAP was elevated in patients with αAQP4+ and concurrent ON when compared with αAQP4- MS [58]. The same group later analyzed GFAP concentration in NMOSD versus RRMS, MS ON, various other ON-opathies and neurological controls, and found that median serum GFAP was significantly higher when compared with most groups, but not neurological controls [59]. Contrary to the previous analysis, they showed that the GFAP levels did not correlate with AQP4 serostatus, even though in the αAQP4+ relapsing isolated ON group, GFAP was significantly higher than in αAQP4- patients [59]. Similarly, in a study done by Fujii and colleagues, GFAP was not different between αAQP4+ and αAQP4- samples [60]. It must be noted, however, that the number of samples per group (*n* = 10 for αAQP4+, *n* = 7 for αAQP4-) were few and for their assay, the serum levels of GFAP were under their limit of detection in > 50% of the samples [60].

Using single-molecule array (SIMOA), Schindler and colleagues found that, although serum GFAP and NFL in αAQP4+ NMOSD had a higher median concentration than in αMOG+ patients and healthy controls, the difference was non-significant [36]. Importantly, αAQP4+ cases with GFAP > 90 pg/mL at baseline had a shorter time to a subsequent attack, hinting at the prognostic value of this marker that previous studies had failed to report [36]. A recent clinical trial corroborated that finding, with serum GFAP showing predictive capacity for future attacks [61]. A 2019 study compared relapse/remission αAQP4+ NMOSD with healthy controls (HC) and relapse/remission RRMS, finding that relapse αAQP4+ NMOSD had significantly higher serum GFAP and NFL in comparison to HC, and higher serum GFAP than remission αAQP4+ NMOSD and relapse/remission RRMS [33]. Finally, studies in CSF have consistently found that GFAP is higher in patients with αAQP4+ and αMOG-/αAQP4- compared to αMOG+ patients and patients with MS or noninflammatory neurological controls; although in some analyses, the highest levels correlated with occurrence of myelitis rather than ON or brain lesions [62–64]. In our analysis, we did not have access to αAQP4+ CSF samples, and thus, we cannot confirm their results.

For NFL, studies have not found large differences in the serum of αAQP4+, αMOG+ and αMOG-/αAQP4- groups. For example, using SIMOA, Lee et al. reported that the levels of NFL in patients with TM did not differ, regardless of the autoantibody titers [65]. Intriguingly, NFL levels correlated with expanded disability status scale (EDSS) scores in the αAQP4+ NMOSD and αMOG+ MOGAD TM groups [65]. Mariotto and colleagues found that serum NFL was significantly higher in αAQP4+ than MS and HC, with a weaker difference between αAQP4+ and αMOG+ and αMOG-/αAQP4- [66]. In CSF, on the other hand, NFL titers were higher in NMOSD compared to MS and other neurological diseases with those values correlating with increased disability [67]. Unfortunately, they did not separate αAQP4+ and αAQP4- cases, thus underutilizing this vital differentiation parameter. In our findings, the αAQP4+ group had a significantly higher NFL concentration than the other groups, but as seen in Fig. 1, the overlap is too large to be of clinical use.

In our analysis, we found that median tau concentration was significantly higher in αMOG-/αAQP4- than in the other groups. Although other significant differences are observable, due to the high intra-group range, there is no clear cut-off distinction between all the other groups. In the literature, serum tau has been evaluated in αMOG+, with results showing that its concentration is higher during relapse than remission [68]. Overall, however, αAQP4+ samples had comparable levels to αMOG+ [68], as seen in our results. Despite this, tau concentrations were able to distinguish αMOG + in the univariate and multivariate logistic regression analysis (Tables 2 and 3), with an AUC = 0.72 (optimism-adjusted; Fig. 3). In parallel, it was integrated in the classification models to produce a more significant result for αAQP4 + classification, with AUC increasing from 0.75 to 0.77 (optimism-adjusted; Fig. 3).

## Limitations

Our study has several limitations, including: (i) lack of definitive diagnosis in the suspected NMOSD samples, (ii) lack of longitudinal follow-up or relapse/remission data, (iii) examining only 3 candidate serum markers, when more molecules could have been added, such as myelin basic protein, S100B, neurofilament heavy chain, etc., (iv) limited number of samples, (v) lack of paired serum with CSF samples to elucidate CNS-periphery correlation.

### Electronic supplementary material

Below is the link to the electronic supplementary material.

Supplementary Material 1: Figure 1. Histogram of the logged distributions of GFAP, NFL and tau in all 86 serum samples.

## Data Availability

The excel file with the concentrations of the three biomarkers and their concentrations are available by request from the corresponding author.

## Abbreviations

αAQP4: autoantibody to aquaporin-4
αMOG: autoantibody to myelin oligodendrocyte glycoprotein
AUC: area under the ROC curve
CNS: central nervous system
NMOSD: neuromyelitis optica spectrum disorder
MOGAD: myelin oligodendrocyte glycoprotein antibody-associated disease
TM: transverse myelitis
ON: optic neuritis
MS: multiple sclerosis
CSF: cerebrospinal fluid
GFAP: glial fibrillary acidic protein
NFL/NfL/NEFL/NF-L: neurofilament light chain
αAQP4+: αAQP4 positive
αMOG+: αMOG positive
αMOG-/αAQP4-: double seronegative for MOG and AQP4
PPMS: primary progressive MS
RRMS: relapsing-remitting MS
PRMS: progressive-relapsing MS
CIS: clinically isolated syndrome
MSD: Meso Scale Discovery
FDR: false discovery rate
BBB: blood-brain barrier
TBI: traumatic brain injury
AD: Alzheimer’s Disease
HC: healthy controls
SIMOA: single-molecule array
EDSS: expanded disability status scale
ROC: receiving operating characteristic
