# Supplementary material for: The relationship between serum astroglial and neuronal markers and AQP4 and MOG autoantibodies
Source: Clin Proteomics. 2024 Apr 5;21:28. doi: 10.1186/s12014-024-09466-9 (PMC10998414; doi:10.1186/s12014-024-09466-9)
Supplement: Supplementary file 1 — Supplementary Material 1: Figure 1. Histogram of the logged distributions of GFAP, NFL and tau in all 86 serum samples. [file 12014_2024_9466_MOESM1_ESM.docx]

**Supplementary Information**


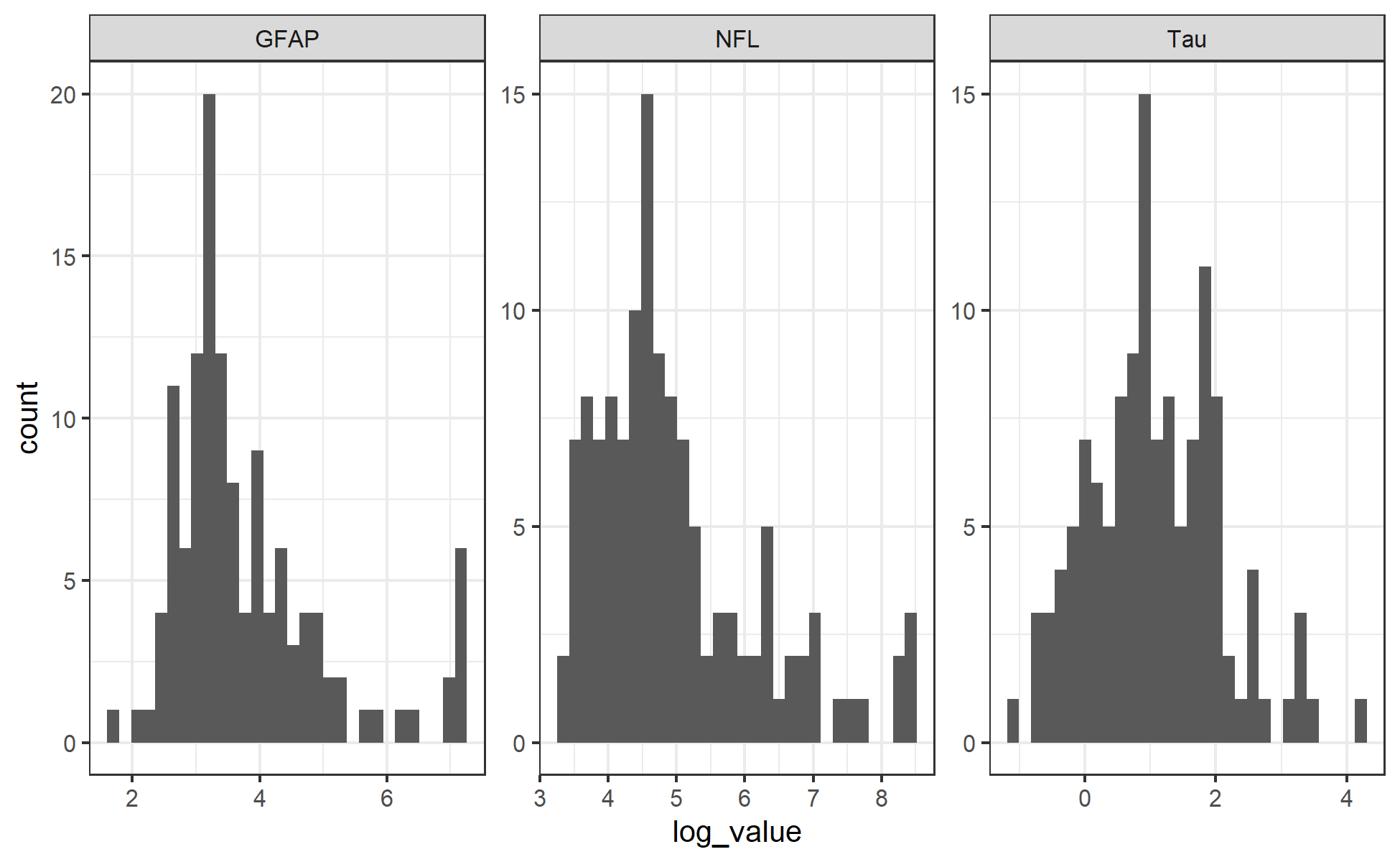


Figure 1. Histogram of the logged distributions of GFAP, NFL and tau in all 86 serum samples.
